# Tuberculous peritonitis in pregnancy: a case report

**DOI:** 10.1186/1752-1947-8-3

**Published:** 2014-01-02

**Authors:** Mounia Lahbabi, Jihane Brini, Khalid Massaoudi

**Affiliations:** 1 Department of Hepato-gastroenterology, Guelmim, Morocco; 2 Department of Obstetrics and Gynecology, Guelmim, Morocco

**Keywords:** Diagnosis, Laparoscopy,Peritoneal tuberculosis, Pregnancy

## Abstract

**Introduction:**

Tuberculous peritonitis is one of the least common forms of extrapulmonary tuberculosis. In the literature, few cases in pregnancy have been previously published. Tuberculous peritonitis in pregnancy is a diagnostic challenge, especially in the absence of lung involvement. It mimics other diseases and clinical presentation is usually non-specific, which may lead to diagnostic delay and development of complications.

**Case presentation:**

We report here a new case of tuberculous peritonitis that occurred in a 31-year-old Caucasian pregnant woman at 22 weeks' gestation. She was complaining of abdominal pain, nausea and vomiting. These symptoms appeared 6 months prior to presentation. Initially, they were attributed to pregnancy, but they progressively became more severe during subsequent weeks. A laparoscopy showed the presence of yellow-white nodules on the peritoneal surface and a biopsy demonstrated caseous necrotic granuloma. She made a good physical recovery after being placed on antituberculous chemotherapy and gave birth to a healthy male neonate of 3100g at 37 weeks' gestation by vaginal delivery.

**Conclusions:**

Extreme vigilance should be used when dealing with unexplained abdominal symptoms to ensure timely diagnosis of tuberculous peritonitis. Diagnosis often requires a histopathological examination. In these patients early diagnosis with early antituberculous therapy are essential to prevent obstetrical and neonatal morbidity.

## Introduction

Peritoneal tuberculosis is one of the least common forms of extrapulmonary tuberculosis [1]. Few cases of tuberculous peritonitis in pregnancy have been recorded. In these patients early diagnosis is important to prevent obstetrical and neonatal morbidity [1]. We report here a new case of tuberculous peritonitis that occurred in a Caucasian pregnant woman at 22 weeks' gestation. The diagnostic and therapeutic problems are discussed, and the relevant literature is briefly reviewed.

## Case presentation

**Figure 1:**
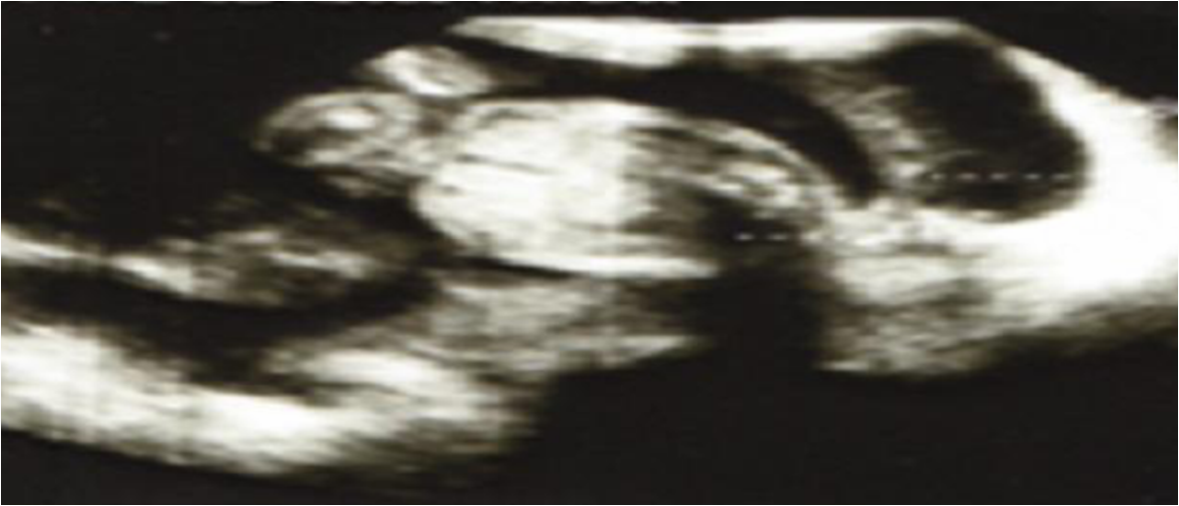
Obstetric ultrasound image showing a pregnancy at 22 weeks of amenorrhea with ascites.

**Figure 2:**
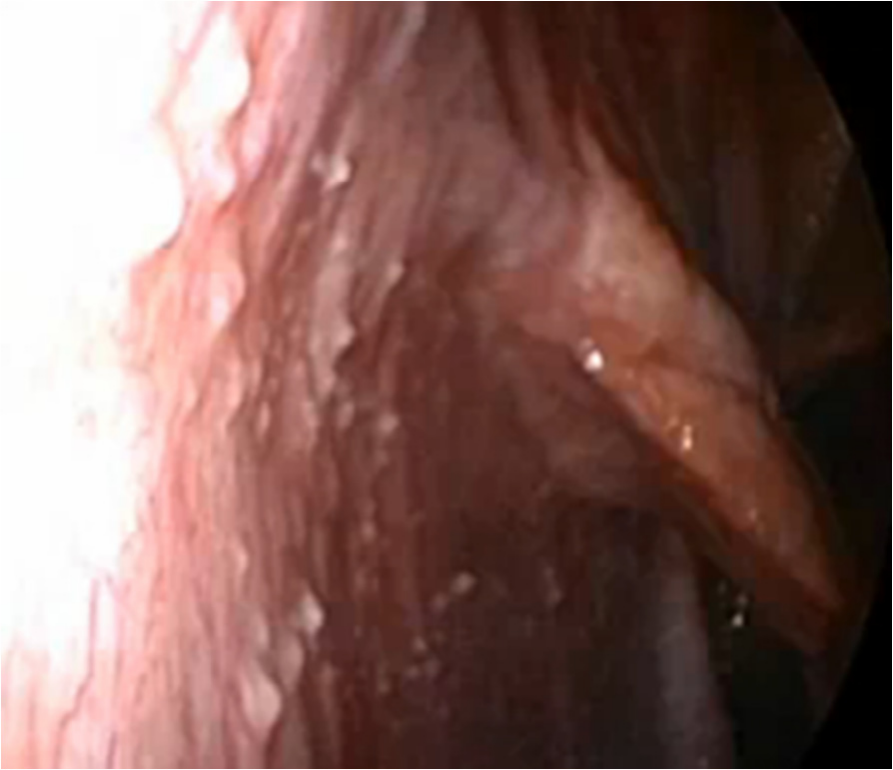
A peritoneal laparoscopy showing multiple extensive yellow-white nodules on the peritoneal surface.

**Figure 3:**
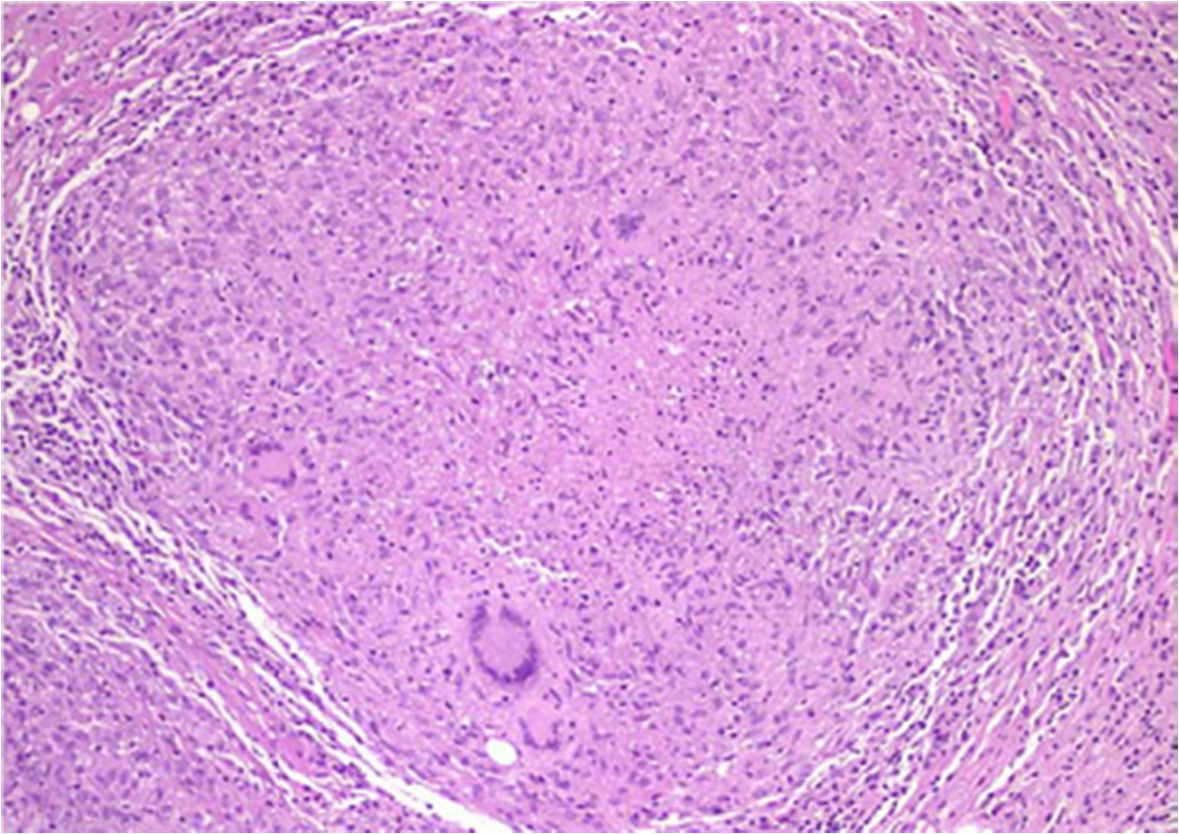
Histological examination demonstrated caseous necrotic granuloma.

A 31-year-old primigravida woman (22 weeks’ pregnant) presented at our Maternity Department complaining of abdominal pain, nausea and vomiting. These symptoms appeared 6 months ago. Initially, they were attributed to pregnancy, but they progressively became more severe during subsequent weeks. A clinical examination revealed a cachectic conscious anicteric woman with mild fever (38°C). The cardiovascular and pleuropulmonary examination were normal. Her abdomen was distended, deep tenderness was elicited in both iliac fossae, and a fluid thrill with shifting dullness confirmed the presence of intraperitoneal free fluid. An abdominal ultrasound confirmed the pregnancy and showed intra-abdominal fluid, mainly in her lower abdomen and a thickened peritoneum (Figure 1). No ovarian mass was identified on ultrasound. A laboratory investigation showed mild normochromic and normocytic anemia (hemoglobin level 10g/dL) and high C-reactive protein without leucocytosis. Her liver function was normal. Serology of viral hepatitis (B and C) and human immunodeficiency virus (HIV) were negative. Of tumor markers, only cancer antigen 125 (CA-125) was found to be high (500U/mL). Ascitic fluid was exudative with a white cell count of 860/mm^3^ (lymphocyte dominant: 480/mm^3^). Her serum-ascites albumin gradient was calculated to be 0.9. Ziehl–Neelsen stain which was investigated in three samples of sputum and in ascitic fluid was negative. The result of a tuberculin skin test was positive. The chest X-ray picture showed no active lesion or old lesion compatible with pulmonary tuberculosis. A diagnostic laparoscopy showed multiple extensive yellow-white nodules on her peritoneal surface with miliary deposits on the intestine (Figure 2), and the biopsy demonstrated caseous necrotic granuloma (Figure 3). She was prescribed antituberculous chemotherapy with rifampicin, isoniazid, and pyrazinamide for 2 months and 4 months of rifampicin-isoniazid. She was given pyridoxine supplementation (25mg/day). After 4 days her general condition improved significantly and her pregnancy continued without any problem. At term a spontaneous vaginal delivery occurred of a live healthy male neonate weighing 3100g. Treatment was well tolerated during pregnancy and after delivery we saw no adverse effects of antituberculosis therapy in either the mother or the neonate.

## Discussion

Peritoneal tuberculosis is an uncommon site of extrapulmonary infection caused by *Mycobacterium tuberculosis*[2]. The risk is increased in patients with cirrhosis, HIV, diabetes mellitus, malignancy, following treatment with anti-tumor necrosis factor and peritoneal dialysis [2]. It is estimated that the incidence of peritoneal tuberculosis among all forms of tuberculosis varies from 0.1% to 0.7% worldwide [3]. Few cases of tuberculous peritonitis in pregnancy have been recorded, suggesting that it is rare [4]. Infection occurs most commonly following reactivation of latent tuberculous foci in the peritoneum that were established from hematogenous spread from a primary lung focus. It can also occur via hematogenous spread from active pulmonary or miliary tuberculosis. Much less frequently, the organisms enter the peritoneal cavity transmurally from an infected small intestine or contiguously from tuberculous salpingitis [5]. The clinical manifestations of tuberculous peritonitis progress insidiously. Pain, fever, chills, weight loss and abdominal pain are common complaints [6]. In pregnant women, diagnosis of tuberculosis may be delayed by the non-specific nature of early symptoms and because they are often attributed to pregnancy [6]. In pregnant women with suggestive symptoms and signs of tuberculosis, a tuberculin skin test should be carried out. This has since been accepted to be safe in pregnancy [7]. A chest radiograph with abdominal lead shield may be done after the tuberculin skin testing, although pregnant women are more likely to experience a delay in obtaining a chest X-ray due to concerns about fetal health [1]. Microscopic examination of sputum or other specimen for acid-fast bacilli remains the cornerstone of laboratory diagnosis of tuberculosis in pregnancy. Three samples of sputum should be submitted for smear, culture, and drug-susceptibility testing [1]. The traditional culture on Lowenstein–Jensen’s medium may take 4 to 6 weeks to obtain a result. This may, however, still be useful in cases of diagnostic doubts and management of suspected drug-resistant tuberculosis [8]. Molecular line probe assay as well as the use of polymerase chain reaction is presently facilitating the specific identification of the tubercle bacilli [9]. As in our patient, the most common clinical presentation of peritoneal tuberculosis is ascites; the fluid is exudate (protein >2.5g/dL) with predomination of mononuclear cells, however 10% of patients may have an initial neutrophilic response [10]. Bacteriologic examination of the ascitic fluid is not always diagnostic: acid-fast smears are rarely positive in tuberculous peritonitis, and conventional cultures yield the pathogen in only 25% of cases [10]. Tuberculous peritonitis may be mistaken for ovarian carcinoma or peritoneal carcinomatosis [2]. Elevation of the serum CA-125 (increased levels indicate ovarian cancer) in pregnancy is not pathognomonic because the serum level of CA-125 can be elevated even in benign diseases including peritonitis [10]. However, the levels of CA-125 have been less than 500U/mL, and it could be used as a follow-up marker in patients treated for peritoneal tuberculosis [10]. The presence of adenosine deaminase activity is also a useful test in the diagnosis: levels above 33U/L are 100% sensitive and 95% specific to the diagnosis [1]. The sensitivity of a computed tomography (CT) scan in the prediction of tuberculosis is 69%. Patients with tuberculosis were likely to show mesenteric changes, macronodules (>5mm in diameter), splenomegaly, and splenic calcification on CT imaging [4]. Accurate diagnosis requires histopathological examination following image-guided biopsy, laparotomy or laparoscopy [11]. Bacteriologic examination of the biopsy specimen should be performed, because this could be positive for tuberculosis when histological examination is negative [11,12]. For the treatment of tuberculosis in pregnant women, the initial regimen should be isoniazid, rifampin, and ethambutol for at least 6 months. Although teratogenicity data for pyrazinamide are limited, it is probably safe to use in pregnancy [2]; in this case the treatment was well tolerated during pregnancy and after delivery and we saw no adverse effect of antituberculosis therapy in the mother or in the neonate. Breastfeeding should not be discouraged for women receiving antituberculosis treatment. Pyridoxine supplementation (25mg/day) is recommended for all pregnant and breastfeeding women taking isoniazid [2].

## Conclusions

Tuberculous peritonitis in pregnancy is a diagnostic challenge, especially in the absence of lung involvement. It mimics other diseases and clinical presentation is usually non-specific, which may lead to diagnostic delay and development of complications. Extreme vigilance should be used when dealing with unexplained abdominal symptoms to ensure timely diagnosis of tuberculous peritonitis. Diagnosis often requires a histopathological examination. Early diagnosis with early antituberculous therapy are essential to prevent obstetrical and neonatal morbidity.

## Consent

Written informed consent was obtained from the patient for publication of this case report and accompanying images. A copy of the written consent is available for review by the Editor-in-Chief of this journal.

## Abbreviations

CA: Cancer antigen; CT: Computed tomography; HIV: Human immunodeficiency virus.

## Competing interests

The authors declare that they have no competing interests.

## Authors’ contributions

LM, MK and BJ analyzed and interpreted the patient data and wrote the manuscript. All authors have read and approved the final manuscript.
